# Association of Insulin Resistance with Bone Strength and Bone Turnover in Menopausal Chinese-Singaporean Women without Diabetes

**DOI:** 10.3390/ijerph15050889

**Published:** 2018-04-30

**Authors:** Maria Kalimeri, Francesca Leek, Nan Xin Wang, Huann Rong Koh, Nicole C. Roy, David Cameron-Smith, Marlena C. Kruger, Christiani Jeyakumar Henry, John J. Totman

**Affiliations:** 1 A*STAR-NUS Clinical Imaging Research Centre, 14 Medical Drive, Singapore 117599, Singapore; ches.leek@gmail.com (F.L.); john_totman@circ.a-star.edu.sg (J.J.T.); 2 Clinical Nutrition Research Centre, 14 Medical Drive, Singapore 117599, Singapore; wangnanxin@nus.edu.sg (N.X.W.); Koh_Huann_Rong@sics.a-star.edu.sg (H.R.K.); jeya_henry@sics.a-star.edu.sg (C.J.H.); 3 Food Nutrition & Health Team, AgResearch Grasslands, Palmerston North 4442, New Zealand; nicole.roy@agresearch.co.nz (N.C.R.); d.cameron-smith@auckland.ac.nz (D.C.-S.); 4 High-Value Nutrition, National Science Challenge, Liggins Institute, The University of Auckland, Auckland 1142, New Zealand; 5 Riddet Institute, Massey University, Palmerston North 4442, New Zealand; M.C.Kruger@massey.ac.nz; 6 Liggins Institute, The University of Auckland, Private Bag 92019, Victoria Street West, Auckland 1142, New Zealand; 7 School of Food and Nutrition, Massey Institute of Food Science and Technology, Massey University, Palmerston North 6420, New Zealand

**Keywords:** insulin resistance, HOMA-IR, DXA, QCT, BMD, Composite Strength Indices, parathyroid hormone (PTH), 25(OH) Vitamin D_3_, CTx-1

## Abstract

Insulin resistance (IR) is accompanied by increased areal or volumetric bone mineral density (aBMD or vBMD), but also higher fracture risk. Meanwhile, imbalances in bone health biomarkers affect insulin production. This study investigates the effect of IR on proximal femur and lumbar spine BMD, femoral neck bending, compressive and impact strength indices (Composite Strength Indices) and circulating levels of parathyroid hormone (PTH), C-telopeptide of Type I collagen (CTx-1) and 25(OH) Vitamin D_3_, in a cohort of 97 healthy, non-obese, menopausal Chinese-Singaporean women. Lumbar spine aBMD was inversely associated with IR and dependent on lean body mass (LBM) and age. No such associations were found for vBMD of the third lumbar vertebra, aBMD and vBMD of the proximal femur, or circulating levels of PTH, CTx-1 and 25(OH) Vitamin D_3_. Composite Strength Indices were inversely associated with IR and independent of LBM, but after adjusting for fat mass and age, this association remained valid only for the impact strength index. Composite Strength Indices were significantly lower in participants with a high degree of IR. Our findings on IR and Composite Strength Indices relationships were in agreement with previous studies on different cohorts, but those on IR and BMD associations were not.

## 1. Introduction

The relationship between Type 2 diabetes mellitus (T2DM) and bone strength—a product of bone mineral density (BMD), geometry and architecture—is complex. People with T2DM have higher BMD, compared to people without the condition, but they also have increased risk of bone fractures [1]. Considering the rise of T2DM globally [2], and the fact that bone fractures cause high rates of morbidity and mortality [3], a better understanding of the relationship between T2DM and bone strength is an important step toward preventing fragility fractures as a complication of T2DM.

Studies suggest that insulin resistance (IR), one of the main drivers of T2DM, plays a direct role in the increased BMD of people with this condition [4,5]. Insulin, a hormone secreted by the pancreas, is involved in the absorption of glucose by cells. Insulin resistance leads to inefficient use, and thus elevated circulating levels, of insulin. It has been proposed that insulin has anabolic effects on bone [6] and that, in turn, bone may play a role in the regulation of insulin sensitivity [7]. On the one hand, osteoblasts, bone cells responsible for the synthesis and mineralization of bone, express the insulin receptor which, when activated by insulin, triggers bone resorption [8]. On the other hand, deletion of the insulin receptor in osteoblasts has been shown to result in insulin resistance and obesity, and osteocalcin, a protein secreted by osteoblasts, promotes insulin synthesis and can regulate insulin sensitivity [9]. Meanwhile, disruption of bone remodeling pathways also affects insulin sensitivity. For example, changes in parathyroid hormone (PTH) levels, a hormone involved in bone remodeling, and Vitamin D deficiency, an important nutrient in bone metabolism, affect the synthesis and release of insulin [7]. Biomarkers of bone formation (P1NP, osteocalcin) and bone resorption (C-telopeptide of Type I collagen-CTx-1) are lower in people with T2DM, compared to people without the condition [5]. The association of body mass index (BMI) and fracture risk has been shown to depend on BMD [10], while high BMI, common in people with IR, has been associated with high BMD. This increase is proportional to lean body mass (LBM) rather than fat mass (FM) or total body mass [11,12], as the dynamic stresses applied on the bones by muscle, but not the static load of fat mass, are thought to stimulate bone formation. Furthermore, studies on cohorts that differ in age, gender and ethnicity, reproductive stages in women (pre-, peri- or post-menopause) and use of diabetes medications have led to varying conclusions regarding the relationship between IR and BMD [13,14]. 

Studies of the effect of IR on the strength of the femoral neck, described by Composite Strength Indices [15], have shown an inverse association of femoral neck strength and insulin resistance [13,16]. Femoral neck strength has also been shown to depend on FM, rather than LBM, in women with non-insulin-requiring T2DM, while LBM, and not FM, was a predictor in a group of men with the same characteristics [17].

The aim of this study was to investigate the effect of IR on bone mineral density of the proximal femur and the lumbar spine, as well as on femoral neck strength, CTx-1, PTH and 25(OH) Vitamin D_3_ concentration levels in healthy, non-obese, menopausal Chinese-Singaporean women. We examined areal and volumetric BMD (aBMD and vBMD, respectively) of the proximal femur and the lumbar spine. In order to examine if IR affects each bone component differently, both whole-bone and compartment-specific vBMD of the proximal femur were measured. Based on previous findings from literature, we hypothesized that bone mineral density is positively associated with IR, while femoral neck strength and bone turnover are inversely associated with IR.

## 2. Materials and Methods

### 2.1. Cohort Characteristics

A total of 97 healthy, non-obese menopausal Chinese-Singaporean women were examined. The participants were between 55 and 70 years of age, at least 5 years menopausal, based on cessation of menstruation (spontaneous or otherwise), and had BMI between 18 and 28 kg/m^2^. None of the participants had been previously diagnosed with osteoporosis or other bone disease, diabetes or any medical condition which affects bone and liver metabolism. The participants did not smoke or drink more than 2 units of alcohol daily and were not taking medications that could affect bone health. The study was given ethical approval by the National Healthcare Group (Singapore), indexed in www.clinicaltrials.gov (NCT 03309254) and all participants provided informed written consent.

### 2.2. Anthropometric Data

The participants reported their age and years since menopause. The height (m), weight (kg), waist and hip circumference (cm) were measured and the BMI was calculated as the ratio of weight and squared height (kg/m^2^).

### 2.3. Biochemical Measurements

Blood and urine samples were collected from the participants after an overnight 12-h fast. Fasting and post-prandial blood samples that were collected from the test session were centrifuged at 1500× *g* for 10 min, and plasma aliquots were sent to National University Hospital, Singapore, Referral Laboratory for insulin and glucose measurements. Insulin was measured using the Cobas e411 (Roche Diagnostics, Switzerland). Glucose was measured using a photometric assay, hexokinase method. Plasma samples were also frozen, stored in dry ice and sent to Massey University, New Zealand. From there, the frozen samples were forwarded to Canterbury Health Laboratories, New Zealand, for analysis. Parathyroid hormone (PTH) and C-telopeptide of Type I collagen (CTx-1) were analyzed by electrochemiluminescence immunoassay using the Roche COBAS^®^ e411 system (Roche Diagnostics, Indianapolis, IN, USA). 25 (OH) Vitamin D_3_ was analyzed using isotope-dilution liquid chromatography-tandem mass spectrometry (ID-LC-MS-MS), as an indicator of Vitamin D status.

The gold standard method for measuring insulin resistance is the glucose clamp technique [18], which is costly, invasive and time-consuming. In this study, the homeostasis model assessment of insulin resistance (HOMA-IR) [19] was used to assess the level of insulin resistance from fasting insulin (FI) and fasting glucose (FG) levels, (FI [mIU/L]·FG [mmol/L]/22.5). HOMA-IR is strongly associated with the glucose clamp technique [20] and higher values indicate higher insulin resistance [21].

The fasting blood glucose of one participant was outside the normal range (>7 mmol/L) and data acquired was thus excluded from further analysis. Unless otherwise stated, data from 96 participants was available.

### 2.4. Body Composition and Areal Bone Mineral Density (aBMD)

Body composition (percentage of body fat, FM and LBM) and areal bone mineral density (aBMD) of the lumbar spine (L_1_–L_4_), the femoral neck and total hip of the non-dominant leg were measured by means of dual X-ray absorptiometry (DXA, Hologic Discovery QDR 4500A densitometer, Hologic Inc. Bedford, MA, USA). 

### 2.5. Volumetric Bone Mineral Density (vBMD)

Volumetric quantitative computed tomography (QCT) of both proximal femurs and the third lumbar vertebra (L_3_) was performed in a clinical CT scanner (Siemens mCT, Erlangen, Germany) using a commercial phantom (Mindways Software Inc., Austin, TX, USA). Peak kilovoltage was 120 kVp and tube current was 200 mAs for the proximal femurs and 100 mAs for L_3_. All scans were analyzed using the phantom manufacturer’s standard software (QCT Pro, version 5.0). vBMD of the trabecular bone compartment of L_3_ was measured, as well as of the whole bone and of the different compartments (cortical and trabecular) of the femoral neck and the total hip of the non-dominant leg. Femoral neck width (FNW), hip axis length (HAL) and DXA-equivalent aBMD of the whole bone and the separate bone compartments of the femoral neck and the total hip were calculated from projections of the proximal femur. The ratio of cortical bone to whole bone in the proximal femur was calculated from the QCT measurements during analysis. Volumetric BMD measurements of the proximal femur were invalid for one of the participants, due to incorrect automatic placement of the regions of interest. As a result, QCT data was available for 95 participants.

### 2.6. Femoral Neck Strength

The strength of the femoral neck was assessed using Composite Strength Indices, a set of indices that take into account aBMD, geometric characteristics of the proximal femur (FNW, HAL) and anthropometric characteristics of the participant (weight, height), to estimate femoral neck strength under different loads as follows [15].

$$\mathrm{Compression}\;\mathrm{strength}\;\mathrm{index}\;\mathrm{CSI}\;=\;\frac{\mathrm{aBMD}\;\cdot \;\mathrm{FNW}}{\mathrm{Weight}}$$

$$\mathrm{Bending}\;\mathrm{strength}\;\mathrm{index}\;\mathrm{BSI}\;=\;\frac{\mathrm{aBMD}\;\cdot {\;\mathrm{FNW}}^{2}}{\mathrm{HAL}\;\times \;\mathrm{Weight}}$$

$$\mathrm{Impact}\;\mathrm{strength}\;\mathrm{index}\;\mathrm{ISI}\;=\;\frac{\mathrm{aBMD}\;\cdot \;\mathrm{FNW}\times \mathrm{HAL}}{\mathrm{Height}\;\times \;\mathrm{Weight}}$$

All three indices were recorded in units of $\frac{g}{\mathrm{kg}\cdot m}$. The compression strength index (CSI) is a measure of the ability of the femoral neck to withstand compressive load in the axial dimension, the bending strength index (BSI) is a measure of its ability to withstand bending forces and ISI is a measure of its ability to absorb the energy of a fall from standing height. FNW and HAL were unavailable for two participants, for which Composite Strength Indices were not calculated. In total, sets of Composite Strength Indices for 94 participants were available. 

### 2.7. Statistical Analysis

Statistical analysis was performed using SPSS for Windows, version 24.0 (SPSS Inc., Chicago, IL, USA). The values are presented in terms of average value and standard deviation. 

Linear relationships between insulin resistance and bone strength, as well as biomarkers in blood, were assessed by means of univariate regression analysis between HOMA-IR and bone-related variables over the entire cohort. The distribution of HOMA-IR values is not normal, thus its log-transformed values were used for analysis instead. As discussed earlier, LBM has been shown to affect BMD and FM has an effect on Composite Strength Indices, while age has also a negative effect on bone strength. In order to isolate the effect of insulin resistance from these parameters, LBM and age were also added to the regression model, and in the case of Composite Strength Indices, models that include FM in the place of LBM were also examined.

Subsequently, the participants were divided into three groups according to HOMA-IR, using the cut-off values reported in [22]. These cut-off values were calculated using data from a cohort of Chinese menopausal women, to distinguish between women with healthy glucose metabolism (HOMA-IR < 1.37), dysglycemia (1.37 ≤ HOMA-IR < 1.97) and T2DM (HOMA-IR ≥ 1.97). Since the cohort in the present study did not include participants with T2DM, these cut-off values were used in our analysis to separate the participants into groups with different levels of insulin resistance. Two-tailed t-tests showed no statistically significant differences between the bone-related parameters in the two groups with higher insulin resistance (HOMA-IR ≥ 1.37). Thus, participants belonging to these groups were pooled into one “high HOMA-IR” group and two-tailed t-tests were performed between the “low HOMA-IR” (HOMA-IR < 1.37) and “high HOMA-IR” groups, to look for potential inter-group differences in aBMD and vBMD of the lumbar spine, aBMD, vBMD, cortical to total bone ratio in the proximal femur, as well as in the Composite Strength Indices of the femoral neck and bone turnover markers. Values of *p* < 0.05 were considered significant. Analysis was performed on the available data of each parameter.

## 3. Results

### 3.1. Cohort Characteristics

After combining the two groups with higher HOMA-IR, 60 participants belonged in the “low HOMA-IR” group and 36 in the “high HOMA-IR” group. The average age, height and years since menopause did not differ significantly between the two groups, but weight, LBM, FM and BMI did (*p* < 0.01) (Table 1). 

### 3.2. Associations between Bone Strength Parameters and Homeostasis Model Assessment of Insulin Resistance (HOMA-IR) 

The results of univariate and multivariate linear regression analysis, in terms of standardized β-coefficients and significance level, for log_10_(HOMA-IR) and R-squared of the model are shown in Table 2. Univariate linear regression analysis resulted in no statistically significant relationship between HOMA-IR and aBMD (DXA and QCT), vBMD (total, cortical and trabecular) of the proximal femur, trabecular bone vBMD of L_3_ or the cortical to total bone volume ratio in the proximal femur. No statistically significant relationships were observed between HOMA-IR and CTx-1, PTH or 25(OH) Vitamin D_3_ levels, either. However, the relationships between the Composite Strength Indices and HOMA-IR were statistically significant; HOMA-IR could be attributed with 13% of the variation in CSI, 6.8% of the variation in BSI and 15.8% of the variation in ISI in the femoral neck.

The relationships between HOMA-IR and Composite Strength Indices remained significant after LBM was added to the regression model. This model explains 14.3%, 8.3% and 16.2% of the variation in CSI, BSI and ISI, respectively. The relationship between DXA aBMD in the lumbar spine and HOMA-IR became stronger (*p* = 0.05); this model explains 17.2% of the variation in lumbar spine aBMD. The relationship between HOMA-IR and trabecular vBMD of L_3_ was strengthened as well, although the result is still not statistically significant (*p* = 0.087).

Adding age as a third independent variable to the model resulted in a statistically significant relationship between lumbar spine aBMD and HOMA-IR. The relationship of HOMA-IR and Composite Strength Indices of the femoral neck remained significant. This model explains 18.9% of the variation in lumbar spine aBMD and 18.6%, 11.2% and 19.7% of the variation in CSI, BSI and ISI, respectively. The relationship between HOMA-IR and trabecular vBMD of L_3_ was strengthened, although the result was still not statistically significant (*p* = 0.075).

Adjusting for FM instead of LBM resulted in a loss of significance for the relationship between HOMA-IR and Composite Strength Indices, although the association of ISI with HOMA-IR was marginally significant (*p* = 0.052). Significance was achieved only for ISI after further adjusting for age (*p* = 0.039); this model explains 29.5% of the variation in ISI.

### 3.3. Comparison of Bone Strength Parameters between “High HOMA-IR” and “Low HOMA-IR” Groups

The average values and standard deviations of the examined parameters of each group and the p-values of two-tailed t-tests between the two HOMA-IR groups are shown in Table 3. aBMD in the lumbar spine and trabecular vBMD in the L_3_ vertebra was lower in the “high HOMA-IR” group, but the differences were only marginally significant (*p* < 0.1). No statistically significant differences were observed in the aBMD, vBMD or cortical to total bone volume ratio of the proximal femur between the two groups. Participants in the “high HOMA-IR” group had lower values of CSI (*p* < 0.01), BSI (*p* < 0.05) and ISI (*p* < 0.01) in the femoral neck. None of the blood biomarkers examined showed statistically significant differences between the two groups, although CTx-1 was higher in the “high HOMA-IR” group (*p* = 0.067). Considering that the association between CTx-1 and HOMA-IR appears to be statistically insignificant in univariate and multivariate linear regression analysis (Table 2), the marginal statistical significance in CTx-1 concentration between the two groups implies that this association is stronger in the separate groups than in the whole cohort. Indeed, univariate regression analysis with log_10_HOMA-IR as the independent variable showed marginally significant associations between HOMA-IR and CTx-1 in the “low HOMA-IR” group (*p* = 0.068, stand. beta coefficient = −0.237) and the “high HOMA-IR” group (*p* = 0.088, stand. beta coefficient = 0.015), which means that an increase of one standard deviation in log_10_HOMA-IR, will lead to a decrease of 0.237 standard deviations in the concentration of CTx-1 in the “low HOMA-IR” group, and to a minor increase of 0.015 standard deviations in the “high HOMA-IR” group.

## 4. Discussion

This is the first study to investigate the association of insulin resistance with bone strength and bone turnover in healthy, non-obese, menopausal Chinese-Singaporean women. The proximal femur and the lumbar spine were examined, as these are two anatomical sites at high risk of osteoporotic fractures. aBMD and vBMD of these sites and Composite (compressive, bending and impact) Strength Indices of the femoral neck were investigated as measures of bone strength, while PTH, CTx-1 and 25(OH) Vitamin D_3_ were examined as blood biomarkers related to bone health. vBMD was measured for the whole bone, as well as its separate components, so as to examine the possible effect of IR in each one. The HOMA-IR index was used for the calculation of insulin resistance. A statistically significant, negative relationship of HOMA-IR and lumbar spine (L_1_–L_4_) aBMD was observed after controlling for LBM and age. No significant associations were observed between HOMA-IR and aBMD or vBMD in the proximal femur, but a statistically significant, negative association was observed between HOMA-IR and all Composite Strength Indices of the femoral neck. Although these relationships remained largely unaffected by LBM and age, significance was lost for the compressive and bending strength indices, but not for the impact strength index, after controlling for FM and age. PTH, CTx-1 and 25(OH) Vitamin D_3_ were not associated with HOMA-IR in the models used. All Composite Strength Indices were significantly lower in the “high HOMA-IR” group, compared to the “low HOMA-IR” group.

The relationship between insulin resistance and BMD has been studied in different populations with mixed results. In a study of menopausal, Caucasian women without diabetes [14], aBMD in the total hip and the lumbar spine showed a statistically significant, positive correlation with HOMA-IR, which disappeared after adding body weight to the model. Meanwhile, in a diverse cohort—relative to age, gender, race, status of diabetes and menopause—after adjusting for those factors and BMI, HOMA-IR was not associated with aBMD of the femoral neck, but was positively associated with lumbar spine aBMD [13]. Finally, in a cohort of South Korean men, HOMA-IR was negatively associated with total body, femoral neck and lumbar spine aBMD, after adjusting for factors such as age, height, weight, percentage of fat mass and lifestyle factors [23]. The primary difference between our study and those mentioned above is the population represented by the cohort. This implies that differences in gender, age and race affect the relationship between insulin resistance and BMD. Of the BMD measurements examined in our study, HOMA-IR was only associated with aBMD in the lumbar spine. The association was negative, and only reached statistical significance after controlling for LBM and age. vBMD of L_3_ was also negatively associated with HOMA-IR in the same regression model, but this relationship was only marginally significant (*p* = 0.075). No statistically significant differences between the aBMD and vBMD measurements of the high- and low- HOMA-IR groups were observed in either anatomical site, although differences in trabecular vBMD of L_3_ were of marginal statistical significance (*p* = 0.098). These results suggest that insulin resistance has a different effect on menopausal Chinese women without T2DM than their Caucasian counterparts, but a similar effect as on the aBMD of Asian men. 

The negative associations observed between HOMA-IR and Composite Strength Indices of the femoral neck were in accordance with those already reported in a number of different cohorts. In [13], the cohort included both men and women of varying ages, different ethnic groups and diabetes status, among others. In [16], the cohort consisted of pre- and peri-menopausal women of diverse ethnic groups and varying diabetes status. Although our study did not include participants with diabetes, all three Composite Strength Indices exhibited a negative association with HOMA-IR and significant differences between the two HOMA-IR groups. Controlling for LBM and age made small difference in the model, which implies that LBM does not affect femoral neck strength. This result is in accordance with the findings in [17], where, in a cohort of women with non-insulin-requiring T2DM, LBM was not a predictor of section modulus and buckling ratio. These parameters describe bending strength and hip cortical stability under compressive load, as measured by means of hip structure analysis [24]. Interestingly, in the aforementioned study, FM was a predictor of the strength parameters in women, while LBM, and not FM, was a predictor in a group of men with the same characteristics. In our study, controlling for FM instead of LBM resulted in a non-significant association between HOMA-IR and Composite Strength Indices; significance was reached only for the impact strength index (ISI), after further controlling for age. These findings indicate that in our cohort, FM, and not LBM, affects the relationship between insulin resistance and femoral neck strength, in accordance with existing literature.

Vitamin D deficiency is known to impair the release and action of insulin [7]. Higher levels of Vitamin D correlated negatively with insulin resistance in a cohort of Chinese men and women with T2DM [25], while raising Vitamin D levels in South Asian women with insulin resistance has been shown to improve insulin sensitivity [26]. Fasting PTH levels have been found to be inversely related to insulin sensitivity, as measured during a hyperglycemic clamp test in healthy, insulin-sensitive individuals [27], while in obese girls, PTH was negatively associated with HOMA-IR [28]. In [4], CTx-1 levels were higher in groups of (both lean and obese) insulin-sensitive people than in groups of obese people with insulin resistance (with or without T2DM). In our study, 25(OH) Vitamin D_3_ (an indicator of Vitamin D status), PTH and CTx-1 concentrations were not significantly associated with HOMA-IR. There were no statistically significant differences in these biomarkers between the high- and low- HOMA-IR groups either. Interestingly, however, the difference in CTx-1 concentrations between the two HOMA-IR groups was marginally significant (*p* = 0.067). This result was explained by the strong associations—still of no statistical significance—between CTx-1 and HOMA-IR in the separate groups. In [4], participants with insulin resistance had lower levels of CTx-1 than insulin-sensitive participants, and CTx-1 concentrations in insulin-resistant states could not be suppressed further. In our study, the “low HOMA-IR” group had lower concentrations of CTx-1 than the “high HOMA-IR” group. The discrepancy between our findings and findings in literature can be attributed to the different profile of our cohort; our participants were all non-obese, menopausal Chinese-Singaporean women without T2DM and in good health. Moreover, since CTx-1 is a bone resorption marker, its lower levels in the “low HOMA-IR” group are in agreement with the negative association between HOMA-IR and aBMD in the lumbar spine.

Our study had several limitations. The participants were separated in groups according to the cut off values reported in [22], which do not necessarily mirror their actual insulin-sensitivity status, of which we have no knowledge. No measurements of osteocalcin and P1NP were available, which would have given an insight into bone formation in relation to insulin resistance. Additionally, only a snapshot of the glycemic status of the participants is available, as fasting levels of glucose and insulin were used for analysis. An oral glucose tolerance test would have given more concrete information about the insulin sensitivity of the participants, while the sampling of blood during a glucose clamp challenge would have given dynamic information about the relationship of blood markers and insulin resistance.

## 5. Conclusions

We have shown that in healthy, non-obese, menopausal Chinese-Singaporean women without Type 2 diabetes melitus, the level of insulin resistance does not affect the bone mineral density of the proximal femur, while the relationship between aBMD of the lumbar spine and insulin resistance is negative and depends on lean body mass and age. Femoral neck strength is inversely related to insulin resistance and is strongly dependent on fat mass, rather than lean mass, and age. Circulating concentrations of 25(OH) Vitamin D_3_, PTH and CTx-1 are not significantly associated with insulin resistance. Femoral neck strength was shown to differ significantly between participants with low and high degrees of insulin resistance, while differences in vBMD of the third lumbar vertebra and CTx-1 are of marginal statistical significance. 

## Figures and Tables

**Table 1:** General characteristics of the total study population, “Low HOMA-IR” and “High HOMA-IR” groups and p values calculated by means of two-tailed t-tests between the two HOMA-IR groups.

	All Subjects			Low HOMA-IR			High HOMA-IR			*p*
N (Group Size)	96			60			36			
**Age (years)**	60.70	±	4.17	60.80	±	4.30	60.50	±	3.99	0.76
**Years menopausal**	10.60	±	6.04	10.20	±	6.00	11.33	±	6.15	0.39
**Weight (kg)**	55.90	±	7.25	54.20	±	6.26	58.78	±	7.96	<0.01*
**Height (m)**	1.56	±	0.05	1.56	±	0.06	1.56	±	0.05	0.94
**Body mass index (BMI) (kg/m^2^)**	22.90	±	2.69	22.20	±	2.44	24.03	±	2.76	<0.01*
**Fat Mass (kg)**	21.90	±	4.96	20.86	±	4.25	23.62	±	5.62	<0.01*
**Lean Body Mass (kg)**	31.59	±	3.10	30.93	±	2.88	32.69	±	3.19	<0.01*
**Fasting glucose (mmol/L)**	4.90	±	0.46	4.80	±	0.41	5.04	±	0.51	<0.05*
**Fasting Insulin (mIU/L)**	6.20	±	3.05	4.40	±	1.21	9.25	±	2.70	<0.01*
**HOMA-IR**	1.36	±	0.71	0.93	±	0.27	2.07	±	0.64	<0.01*

*p*: significance level between Low HOMA-IR group and High HOMA-IR group. * *p* < 0.05.

**Table 2:** Results of univariate and multivariate linear regression analysis between log_10_HOMA-IR and areal BMD (aBMD), volumetric BMD (vBMD), cortical bone ratio, Composite Strength Indices and blood markers.

	Unadjusted			Adjusted for LBM			Adjusted for LBM and Age		
	β	*p*	R^2^	β	*p*	R^2^	β	*p*	R^2^
**DXA aBMD**									
*Femoral neck*	0.054	0.604	0.003	−0.064	0.540	0.113	−0.064	0.531	0.162
*Total hip*	0.000	0.997	0.000	−0.096	0.365	0.075	−0.096	0.359	0.111
*Lumbar spine*	−0.053	0.605	0.003	−0.199	0.050	0.172	−0.199	**0.049**	0.189
**QCT aBMD**									
*Femoral neck*									
total	−0.062	0.549	0.004	−0.144	0.185	0.057	−0.144	0.177	0.097
cortical	−0.047	0.648	0.002	−0.103	0.350	0.027	−0.103	0.346	0.050
trabecular	0.008	0.942	0.000	−0.050	0.645	0.027	−0.051	0.644	0.041
*Total hip*									
total	−0.015	0.888	0.000	−0.097	0.367	0.055	−0.098	0.358	0.095
cortical	−0.044	0.671	0.002	−0.103	0.349	0.029	−0.103	0.337	0.083
trabecular	0.086	0.410	0.007	−0.013	0.899	0.085	−0.013	0.900	0.087
**QCT vBMD**									
*Femoral neck*									
total	−0.046	0.659	0.002	−0.065	0.557	0.005	−0.065	0.555	0.026
cortical	0.085	0.415	0.007	0.088	0.426	0.007	0.088	0.416	0.052
trabecular	−0.019	0.855	0.000	−0.022	0.840	0.000	−0.023	0.837	0.031
cort.vol/tot.vol	−0.093	0.369	0.009	−0.114	0.301	0.012	−0.114	0.293	0.054
*Total hip*									
total	−0.006	0.956	0.000	−0.003	0.976	0.000	−0.003	0.975	0.040
cortical	−0.049	0.636	0.002	−0.107	0.329	0.029	−0.107	0.328	0.047
trabecular	0.040	0.703	0.002	0.023	0.835	0.004	0.023	0.836	0.015
cort.vol/tot.vol	−0.070	0.500	0.005	−0.048	0.666	0.009	−0.048	0.666	0.016
*L_3_ vertebra*									
trabecular	−0.140	0.175	0.020	−0.187	0.087	0.037	−0.187	0.075	0.120
**Composite Strength Indices**									
CSI	−0.360	0.000*	0.130	−0.319	0.003*	0.143	−0.320	0.002*	0.186
BSI	−0.261	0.011*	0.068	−0.219	0.042*	0.083	−0.220	0.039*	0.112
ISI	−0.397	0.000*	0.158	−0.373	0.000*	0.162	−0.374	0.000*	0.197
**Bone Turnover Markers**									
CTx-1	0.071	0.491	0.005	0.064	0.561	0.005	0.064	0.562	0.009
25(OH) Vitamin D_3_	−0.002	0.984	0.000	0.028	0.798	0.007	0.028	0.798	0.024
PTH	0.109	0.291	0.012	0.030	0.780	0.062	0.030	0.780	0.069

LBM: lean body mass, β: standardized β-coefficient, *p*: significance level, R^2^: R-squared of the model; DXA: dual X-ray absorptiometry, QCT: quantitative computed tomography, cort.vol/tot.vol: cortical to total volume ratio; CSI: compression strength index, BSI: bending strength index, ISI: impact strength index, CTx-1: C-telopeptide of Type I collagen, PTH: parathyroid hormone. * *p* < 0.05.

**Table 3:** Average values and standard deviations of parameters in “low HOMA-IR” and “high HOMA-IR” groups and *p*-values of two-tailed *t*-tests between the two groups.

	Low HOMA-IR Group			High HOMA-IR Group			*p*
**DXA aBMD**							
*Femoral neck* (g/cm^2^)	0.64	±	0.09	0.64	±	0.08	0.978
*Total hip* (g/cm^2^)	0.79	±	0.11	0.78	±	0.09	0.536
*Lumbar spine* (g/cm^2^)	0.87	±	0.14	0.85	±	0.13	0.404
**QCT aBMD**							
*Femoral neck*							
total (g/cm^2^ K_2_HPO4)	0.69	±	0.11	0.66	±	0.09	0.160
cortical (g/cm^2^ K_2_HPO_4_)	0.44	±	0.11	0.42	±	0.07	0.299
trabecular (g/cm^2^ K_2_HPO_4_)	0.24	±	0.03	0.24	±	0.04	0.377
*Total hip*							
total (g/cm^2^ K_2_HPO_4_)	0.75	±	0.12	0.72	±	0.10	0.257
cortical (g/cm_2_ K_2_HPO_4_)	0.46	±	0.11	0.43	±	0.08	0.169
trabecular (g/cm^2^ K_2_HPO_4_)	0.28	±	0.03	0.29	±	0.04	0.683
**QCT vBMD**							
*Femoral neck*							
total (mg/cm^3^ K_2_HPO_4_)	312.58	±	56.31	301.46	±	36.53	0.294
cortical (mg/cm^3^ K2HPO4)	982.96	±	113.00	998.28	±	109.29	0.518
trabecular (mg/cm^3^ K_2_HPO_4_)	139.44	±	19.42	134.77	±	19.70	0.261
cortical to total volume ratio	0.21	±	0.07	0.20	±	0.05	0.266
*Total hip*							
total (mg/cm^3^ K_2_HPO_4_)	277.32	±	59.01	270.17	±	37.19	0.516
cortical (mg/cm^3^ K_2_HPO_4_)	926.69	±	85.80	924.89	±	76.49	0.918
trabecular (mg/cm^3^ K_2_HPO_4_)	133.29	±	17.45	131.23	±	17.76	0.580
cortical to total volume ratio	0.22	±	0.24	0.18	±	0.04	0.294
*L_3_ vertebra*							
trabecular (mg/cm^3^ K_2_HPO_4_)	109.08	±	24.55	99.86	±	28.79	0.098
**Composite strength indices**							
CSI (g/(kg×m))	3.56	±	0.61	3.16	±	0.57	0.002*
BSI (g/(kg×m))	0.99	±	0.22	0.89	±	0.19	0.019*
ISI (g/(kg×m))	0.23	±	0.04	0.20	±	0.04	0.001*
**Bone Turnover Markers**							
CTx-1 (µg/L)	0.51	±	0.18	0.60	±	0.30	0.067
25(OH)D3 (nmol/L)	59.03	±	14.65	60.33	±	14.30	0.672
PTH (pmol/L)	4.65	±	1.26	5.24	±	3.30	0.218

*p*: significance level, cort.vol/tot.vol: cortical to total volume ratio; CSI: compression strength index, BSI: bending strength index, ISI: impact strength index. * *p* < 0.05.
